# Very Low Internal Echoes With Enhanced Posterior Echoes Suggest the Aggressive Nature of Expanding Ovoid Breast Cancers: A Case Report

**DOI:** 10.7759/cureus.78014

**Published:** 2025-01-26

**Authors:** Ayaka Kurumada, Shoji Oura, Mariko Honda

**Affiliations:** 1 Department of Surgery, Kishiwada Tokushukai Hospital, Kishiwada, JPN; 2 Department of Surgery, Izumiotsu Municipal Hospital, Izumiotsu, JPN

**Keywords:** breast cancer, enhanced posterior echoes, high ki-67 labelling index, tumor aggressiveness, very low internal echoes

## Abstract

A 40-year-old woman with a left breast mass was referred to our hospital. Mammography showed only a mass shadow. An ultrasound showed a 30mm oval mass with distinct borders, focal cystic parts, enhanced posterior echoes, and very low internal echoes. Magnetic resonance images showed that the tumor had mixed hypo- and hyper-intense areas both on T1- and fat-suppressed T2-weighted images. In addition, time-signal intensity images showed a washout pattern in the solid part of the tumor. A vacuum-assisted biopsy of the mass showed highly atypical cells growing in a solid fashion. The patient, therefore, underwent breast-conserving surgery and sentinel node biopsy. A post-operative pathological study showed that the tumor had a solid growth pattern, intra-tumoral bleeding, a triple-negative phenotype, a nuclear grade of 3, and a Ki-67 labeling index of 90%. Very low internal echo areas were consistent with the proliferative areas of poorly differentiated cancer cells. In conclusion, tumors should be highly aggressive when they have expanding ovoid shapes, enhanced posterior echoes, and very low internal echoes.

## Introduction

Patients with breast cancer have favorable clinical outcomes compared to those with various solid malignancies. Similar to other solid malignancies, the stage of the disease is the most important prognostic factor in breast cancer. Therefore, breast cancer patients with larger tumor sizes and multiple positive nodes are more likely to experience recurrence than those with smaller tumor sizes and no or fewer positive nodes [1-3]. Other important prognostic factors include high nuclear grade [4], vessel involvement [5], estrogen receptor negativity [6], and a high Ki-67 labeling index [7]. However, no clear correlation, except for tumor vascularities [8], between image findings and breast cancer biology has been reported to date.

Herein, we report an oval breast cancer in which the correlation between very low internal echoes with enhanced posterior echoes and breast cancer aggressiveness was pathologically evaluated.

## Case presentation

**Figure 1:**
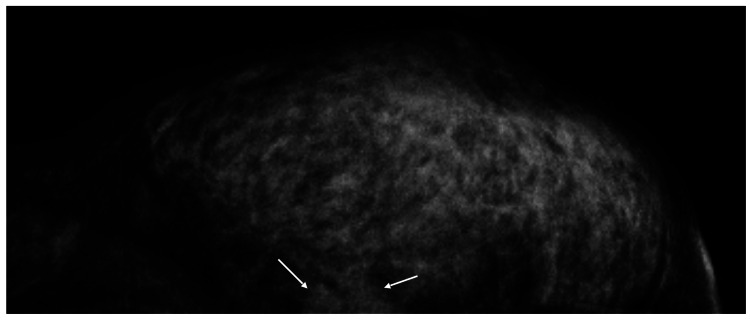
Mammographic findings Mammography showed only a part of the mass shadow (arrows).

A 40-year-old woman with a left breast mass was referred to our hospital. The mammography showed only a part of the mass shadow in the upper inner quadrant of her left breast (Figure 1).

**Figure 2:**
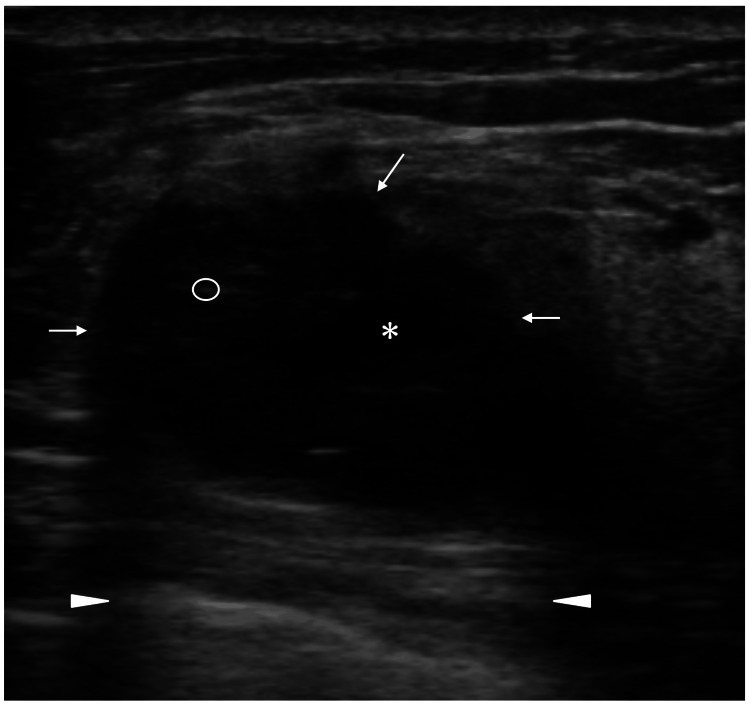
Ultrasound findings Ultrasound showed an oval mass with distinct mass borders (arrows), a focal presumed liquid part (asterisk), faint punctate internal high echoes (circle), enhanced posterior echoes (arrowheads), and very low internal echoes.

Ultrasound (US) revealed a 30mm oval mass with distinct borders, focal cystic parts, punctate internal high echoes in limited areas, enhanced posterior echoes, and very low internal echoes (Figure 2).

**Figure 3:**
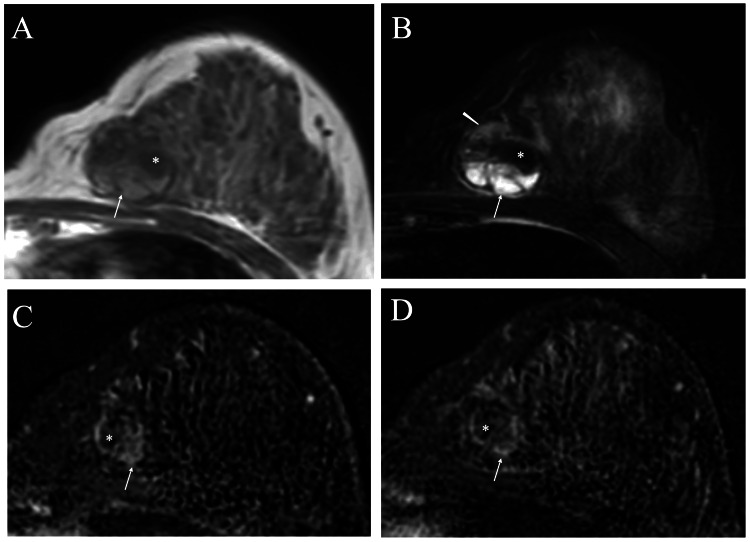
Magnetic resonance image (MRI) findings A. T1-weighted images showed both hypo- (asterisk) and hyper- (arrow) intense areas in the mass. B. Fat-suppressed T2-weighted images showed areas that were hypo-intense (asterisk), slightly hyper-intense (arrowhead), and markedly hyper-intense (arrow). C. Time-signal intensity images showed hypo-intense (asterisk) and hyper-intense (arrow) areas in the mass on early phase images. D. Time-signal intensity images showed that the hypo-intense area (asterisk) remained hypo-intense but the hyper-intense area showed a washout pattern (arrow) on late-phase images.

Magnetic resonance images (MRIs) showed that the tumor had mixed hypo- and hyper-intense areas both on T1- and fat-suppressed T2-weighted images. In addition, time-signal intensity images showed a washout pattern in the solid part of the tumor (Figure 3).

**Figure 4:**
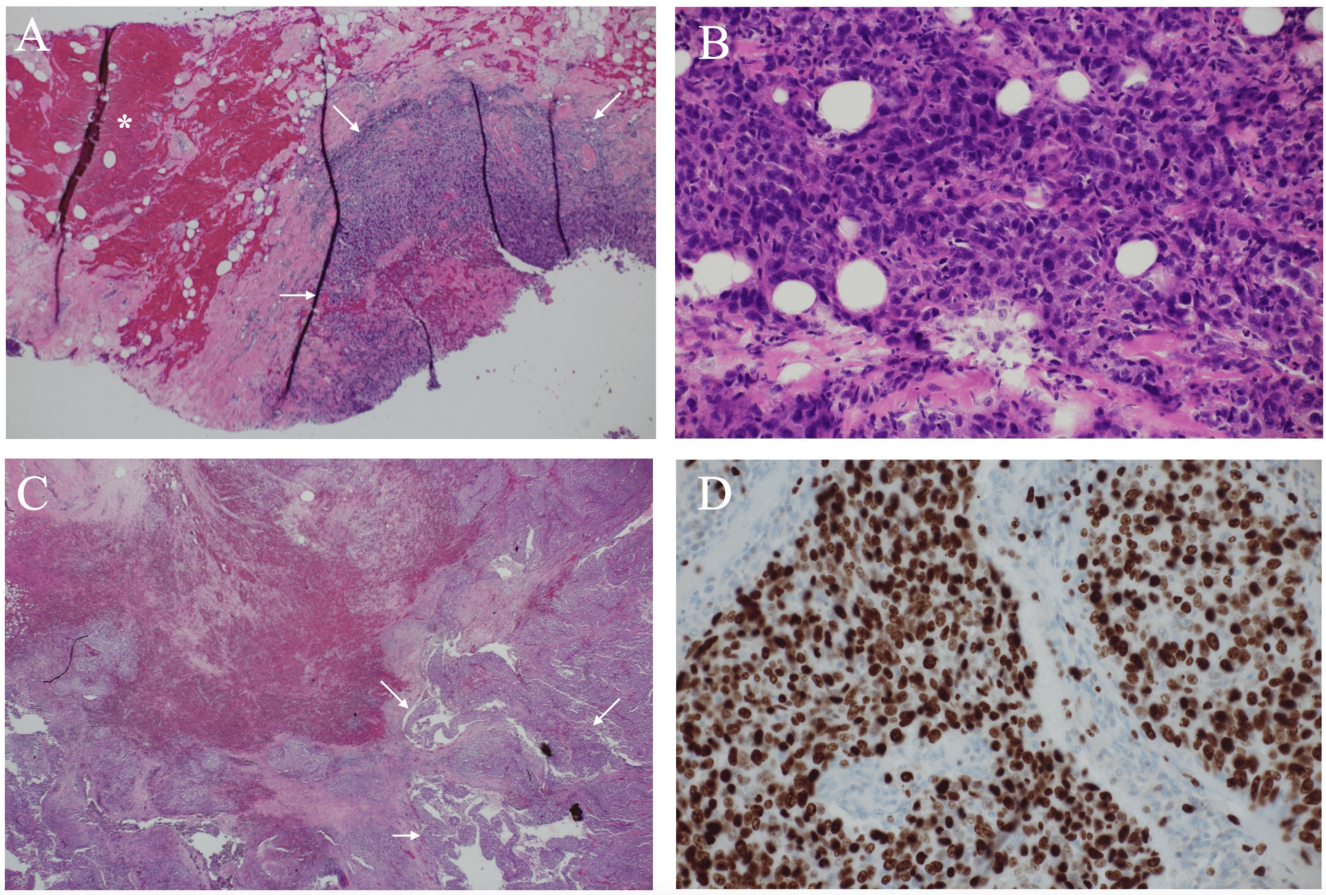
Pathological findings A. Low magnified view of the biopsy specimen showed atypical cells (arrows) and massive bleeding (asterisk). B. Magnified view of the biopsy specimen showed that highly atypical large cells grew in a solid fashion. C. Low magnified view showed that the tumor grew in a solid-papillary growth pattern (arrows) in very limited areas. D. The tumor showed a very high Ki-67 labelling index of 90%.

The patient underwent a vacuum-assisted biopsy (VAB) of the mass under the tentative diagnosis either of breast cancer or phyllodes tumor. VAB was done to the solid part of the tumor and caused simultaneous excretion of dark red liquid on tissue sampling. A pathological study showed highly atypical cells growing mainly in a solid fashion with intra-tumoral bleeding (Figures 4A-4B). Immunostaining showed that the tumor had a triple-negative phenotype and a markedly high Ki-67 labeling index of 80%. As a result, the patient underwent breast-conserving surgery and sentinel node biopsy due to the clinical node negativity. The frozen section showed neither positive margins nor positive sentinel nodes. The post-operative pathological study showed that the tumor had intra-tumoral bleeding and grew mainly in a solid pattern (Figure 4C). Immunostaining of the tumor showed a higher Ki-67 labeling index of 90% and a similar pathological phenotype to that of the VAB specimen (Figure 4D).

The patient recovered uneventfully and received adjuvant chemotherapy followed by radiotherapy to the conserved breast.

## Discussion

Mammography did not show any malignant findings due to location-induced insufficient mass depiction in this case. However, the background-dense breast would have made it difficult to show definitive malignant findings, even if the tumor had been located in easily mass-despicable areas, on mammography. In addition, diagnostic physicians never diagnose a well-circumscribed oval mass, even though having been clearly depicted on mammography, as breast cancer. The MRI provided more cancer-related information than the mammography and suggested that the tumor had blood components such as deoxyhemoglobin and methemoglobin in addition to cancer components. Furthermore, the MRI expressed how densely cancer cells were present in the solid part of the tumor. However, similar to mammography, the MRI did not indicate any findings about tumor aggressiveness except for vascularities in and around the tumor.

It is well known that ultrasound wave reflection and back scattering determine the tumor shape and internal echogenicity, respectively. The heterogeneity of tissue components generates ultrasound wave backscattering due to the different acoustic impedances between the cells or materials in contact and makes the internal echoes of the tumor higher as the difference in acoustic impedances increases [9]. In addition, the presence of some kind of papillary structures also generates ultrasound wave backscattering. Conversely, homogeneity of the mass components generates less ultrasound wave backscattering and therefore makes the internal echoes of the target mass low. Very low internal echoes, therefore, generally imply that the tumor is highly proliferative [10]. In addition to very low internal echoes, round and oval tumor shapes also suggest the tumor's aggressiveness. Aggressive cancer cells generally grow in an expanding manner independently of intra-mammary structures such as mammary ducts and lobules and typically form round or oval tumors. Breast malignant lymphomas and triple-negative breast cancers with BRCA mutation generally have round or oval shapes with very low internal echoes.

In this case, the breast cancer, fortunately, had no lymph node metastasis but had a triple-negative phenotype and a very high Ki-67 labeling index of 90%. Although no consensus on what the cutoff value of Ki-67 should be as an indicator of aggressiveness in breast cancer, 90% of the Ki-67 labeling index is a very high value and strongly suggests that the tumor is highly aggressive.

## Conclusions

It is very important to evaluate the tumor aggressiveness in developing a treatment strategy for breast cancer. Therefore, various prognostic factors, including lymph node metastasis, have been identified for breast cancer. No reports, however, have shown a correlation between tumor shapes / internal echo patterns and tumor aggressiveness to date. It, however, is easy to imagine that high proliferation potential leads to uniform and expansive growth of the tumor. This study indicates that tumors should be highly aggressive when they show expanding-ovoid-type shapes and have very low internal echoes with enhanced posterior echoes.
